# Decreased functional expression of Grp78 and Grp94 inhibits proliferation and attenuates apoptosis in a human gastric cancer cell line *in vitro*

**DOI:** 10.3892/ol.2014.2831

**Published:** 2014-12-24

**Authors:** XINCHEN ZHANG, LIYING ZHANG, SHU WANG, DEQUAN WU, WEILIANG YANG

**Affiliations:** 1 Department of General Surgery, The Second Affiliated Hospital of Harbin Medical University, Harbin, Heilongjiang 150086, P.R. China; 2 Department of Obstetrics and Gynecology, The Second Affiliated Hospital of Harbin Medical University, Harbin, Heilongjiang 150086, P.R. China

**Keywords:** Grp78, Grp94, RNA interference, cell proliferation, apoptosis

## Abstract

The aim of the present study was to determine the effect of downregulating the expression of glucose-regulated protein 78 (Grp78) and Grp94 upon the rate of proliferation and apoptosis in the human gastric cancer SGC-7901 cell line. The SGC-7901 cells were divided into three groups as follows: i) An experimental group co-transfected with the small interfering RNA vectors, psiSTRIKE™/Grp78 and psiSTRIKE/Grp94; ii) a negative control group, in which only Lipofectamine 2000™ was used to transfect the cells; and iii) a blank control group, in which cells were left untouched and not transfected with any agent. The transcriptional expression of Grp78 and Grp94 was assayed by reverse transcription polymerase chain reaction, and the protein expression of Grp78 and Grp94 was determined using an immunofluorescence assay at 24, 48 and 72 h post-transfection. The rates of cellular proliferation and apoptosis were assayed using MTT and flow cytometry analyses, respectively. The mRNA and protein expression of Grp78 and Grp94 in the gastric cancer cells was downregulated at 72 h post-transfection. In addition, the results of the MTT assay revealed that the proliferation rate of the gastric cancer cells in the co-transfected group was significantly inhibited at 72 h post-transfection compared with the control groups (P<0.05). The apoptosis ratio was significantly increased in the experimental group compared with the control groups (P<0.05). The co-transfection of the SGC-7901 cells with psiSTRIKE/Grp78 and psiSTRIKE/Grp94 markedly reduced the expression of Grp78 and Grp94, respectively. Furthermore, the reduction in the expression of Grp78 and Grp94 inhibited cellular proliferation and significantly downregulated the rate of apoptosis in the SGC-7901 cells *in vitro*.

## Introduction

Glucose-regulated proteins (Grps) are a group of constitutively expressed molecular chaperones. In a number of cancer tissues, the expression of Grps is significantly upregulated, which is mediated, in part, by the cellular response to a variety of stressful conditions, such as glucose deprivation, oxidative stress and hypoxia (1). Solid tumors, such as colon and lung cancers, commonly contain regions exposed to glucose deprivation and hypoxia. These conditions favor poor vascularization, which ultimately results in acidosis and alterations in cellular metabolism. The endoplasmic reticulum (ER) responds to such cellular stress by initiating specific signal transduction pathways, which protect the cell against stress-induced apoptosis. Grps are activated as a consequence of accumulated misfolded proteins in the ER and through the unfolded protein response (UPR) pathway (2). The most important and well-studied Grps are Grp78 and Grp94. The expression of Grp78 and Grp94 is a defense mechanism used by cancer cells for survival (3,4).

The overexpression of Grp78 and Grp94 has been associated with a number of malignant tumors. Previous studies have identified a correlation between Grp78 and Grp94 mRNA and protein expression and the stage and behavior of esophageal adenocarcinomas (5–7). The high expression level of Grp78 and Grp94 in advanced stages of cancer may also depend upon other factors, such as glucose deprivation-induced cellular stress pathways, hypoxia or the protective host immune response. Another study revealed that the expression of Grp78 and Grp94 was associated with the differentiation and progression of lung cancer (8). The expression of Grp78 and Grp94 mRNA and protein may therefore be useful for evaluating the differentiation and clinical stage of human lung cancer (9,10). A previous study suggested that high Grp94 protein expression could represent one of the molecular mechanisms that may contribute to the resistance of tumor cells to radiation. Therefore, it was hypothesized that Grp94 small interfering RNA (siRNA) could be clinically useful as a tumor-specific gene therapy to reverse radioresistance in cervical cancers when administered prior to induction radiotherapy (11). A further study identified that high Grp94 expression may represent a molecular mechanism to lower the resistance of cancer cells to Adriamycin (12). Other studies also demonstrated that Grp78 and Grp94 were associated with certain tumors, when expressed either together or alone (13–16).

Based on the results of previous studies, the present study aimed to simultaneously suppress the expression of Grp78 and Grp94 by co-transfection. As Grp94 may represent another important factor involved in the pathogenesis of gastric cancer, it was hypothesized that by downregulating the expression of Grp78 and Grp94 the proliferation of the gastric cell line could be inhibited.

## Materials and methods

### Materials

The gastric cancer SGC-7901 cell line was obtained from the Scientific Research Foundation of the Second Affiliated Hospital of Harbin Medical University (Harbin, China). The Grp78 and Grp94 gene hairpin oligonucleotide sequences, and all primers, were synthesized by Boya Biological Pharmaceutical Co., Ltd. (Fuzhou, China).

### Construction of the siRNA expression vector

The RNA interference recombinant plasmids, specific for Grp78 and Grp94, were constructed and named psiSTRIKE™/Grp78 and psiSTRIKEGrp94, respectively. Following transfection, the expression of Grp78 and Grp94 mRNA and protein was significantly inhibited by psiSTRIKE/Grp78 and psiSTRIKE/Grp94, respectively (17,18).

Subsequent to dilution and annealing, the oligonucleotides were constructed with the psiSTRIKE-puromycin vector (Promega, Fitchburg, WI, USA). The psiSTRIKE-puromycin vector contains a *pst*I enzyme site, which following recombination, separates into two distinct sites. Prior to and subsequent to restriction digestion of the *pst*I incision enzyme, the recombinant vector contains one or two DNA fragments, respectively. The oligonucleotides of the human Grp78 and Grp94 genes, which are used to code sequences, were selected by accessing GenBank. The sense and antisense sequences of the Grp78 and Grp94 siRNA duplexes were as follows: Grp78 sense, 5′-ACCGCAAGAATTGAA ATTGAGTTTCAAGAGAACTCAATTTCAATTCTTGCT TTTTC-3′ and antisense, 5′-TGCAGAAAAAGC AAGAATTGAAATTGAGTTCTCTTGAAACTCAATTTC AATTCTTG-3′; Grp94 sense, 5′-ACCGAGGAAGAA GAAGAAGAAATTCAAGAGATTTCTTCTTCTTCTTCC TCTTTTTC-3′, and antisense, 5′-TGCAGAAAAAGA GGAAGAAGAAGAAGAAATCTCTTGAATTTCTTCTTC TTCTTCCT-3′. The sense and antisense sequences of the scrambled Grp78 and Grp94 siRNA duplexes were follows: Non-Grp78 sense, 5′-ATTAACGTC ACGTGTTAGATAATCGTGAAGTCAACTTAACAGTAA TTCACTTTTTC-3′ and antisense, 5′-TGCAGAAATTAA CGTCACGTGTTAGATAATCGTGAAGTCAACTTAACAGT AATTCA-3′; non-Grp94 sense, 5′-GTAGTTAATAGG TATACCTAGCCATGCAATGAACATATAGTCATCGATCCT TTTTC-3′ and antisense, 5′-TGCAGAAGTAGTTAA TAGGTATACCTAGCCATGCAATGAACATATAGTCAT CGATC-3′.

### Cell culture and transfection

The human gastric cancer SGC-7901 cell line was maintained in Dulbecco’s modified Eagle’s medium (DMEM; Gibco, Rockville, MD, USA), supplemented with 10% fetal bovine serum (Gibco) containing 100 U/ml penicillin and 100 U/ml puromycin in 5% CO_2_ humidified air. The cells were cultured at a density of 3×10^5^ cells per well in 6-well plates, and then divided into the experimental group, the negative control group and the blank control group. Following a 24-h incubation period, the cells in the experimental group were transfected, using the Lipofectamine 2000™ reagent, with 4 μg siRNA expression plasmid. In total, 4 μg scrambled sequence siRNA plasmid was transfected into the cells of the negative control group, and 4 μg psiSTRIKE-puromycin vector was transfected into the cells of the blank control group. Following a further 24-h incubation, the cells were trypsinized and one well of the treated cells was distributed among five 100-mm petri dishes. The culture medium was replaced every three days with culture medium containing 1 mg/ml puromycin until clones had formed that were large enough to isolate (after two to three weeks).

### Semi-quantitative reverse transcription polymerase chain reaction (RT-PCR)

The Grp78 and Grp94 total mRNA (500 ng) was extracted using TRIzol reagent (Invitrogen Life Technologies, Carlsbad, CA, USA) from the cells of the treated experimental group and the negative control group. The mRNA concentration and optical density (OD)_260_ / OD_280_ ratios (>1.8) were determined by ultraviolet photospectrometry (Gel Doc XR+; Bio-Rad Laboratories Inc., Hercules, CA, USA) The total RNA (1 μg) was reverse-transcribed into cDNA and then expanded by PCR using the Stratagene Mx3000P qPCR system (Agilent Technologies, Inc., Santa Clara, CA, USA) and the KAPA SYBR^®^ FAST qPCR kit (Kapa Biosystems Inc., Wilmington, MA, USA). The primer sequences for Grp78, Grp94 and β-actin were as follows: Grp78 primer 1, 5′-TGGGTCGACTCGAATTCCAAAG-3′ and primer 2, 5′-GTCAGGCGATTCTGGTCATTGG-3′; Grp94 primer 1, 5′-ACTGTTGAGGAGCCCATGGAGG-3′ and primer 2, 5′-GCTGAAGAGTCTCGCGGGAAAC-3′; and β-actin primer 1, 5′-CTGCAATCCGAAAGAAGCTG-3′ and primer 2, 5′-ATCTTCAAACCTCCATGATG-3′. The PCR conditions were as follows: 48°C for 50 min, 94°C for 2 min, 94°C for 30 sec, 56°C for 40 sec, 70°C for 80 sec, 35 cycles at 70°C for 7 min and a final cool down to 4°C.

The PCR products were analyzed using 1.5% agarose gel electrophoresis, and images were captured using Kodak Image Station 4000MM (Kodak, Rochester, NY, USA). The ratio of Grps to β-actin was used to compare the relative levels of Grp mRNA. The PCR was repeated three times. The relative levels of Grp mRNA were determined as follows: Grp mRNA level = OD of sample / OD of β-actin.

### Indirect immunofluorescence assay

The cells of all groups, which were grown on glass slides, were rinsed three times in phosphate-buffered saline (PBS) and then fixed in either methanol at −20°C for 4 min or 3.5% formaldehyde at room temperature for 7 min. Following fixation, the cells were permeabilized in 0.05% Tween 20 for 5 min, washed three times in PBS and then processed for indirect immunofluorescence. The SGC-7901 cells were labeled with CellTracker™ Blue CMAC (Invitrogen Life Technologies) and then mixed with equal numbers of anergic or *in vitro-*primed cells. After ~8 min, the cells were fixed and made permeable prior to staining with mouse anti-human monoclonal anti-GRP78, anti-GRP94 and anti-talin antibodies (1:50; Santa Cruz Biotechnology, Inc., Santa Cruz, CA, USA). Next, goat anti-mouse secondary antibodies (1:100 dilution; Santa Cruz Biotechnology, Inc.), conjugated to fluorescein isothiocyanate (FITC) or phycoerythrin, were added. The cells were analyzed using a Zeiss Axiovert 100 microscope (Zeiss, Oberkochen, Germany), and 15 conjugates were typically assigned one of the following scores: 1, <25% fluorescence intensity; 2, 25–50% fluorescence intensity; 3, 51–75% fluorescence intensity; 4, >75% fluorescence intensity. Image capture and deconvolution analysis were performed using SlideBook software (Intelligent Imaging Innovations, Inc., Denver, CO, USA). Subsequent to staining, the glass slides were washed in PBS and then mounted in Gelvatol containing 100 mg/ml DABCO (Sigma-Aldrich, St. Louis, MO, USA). The images of fixed, stained preparations were captured using a Zeiss LSM 510 microscope (Zeiss). The positive cells from the experimental group, identified following transfection, were compared with the cells from the negative control group and with the untransfected cells. In total, three images were used from each of the selected time-points. In addition, 200 cells were observed in each of the images captured by a ×40 objective lens for each field of view. The positive cells and the rates of positive cell staining were counted.

The cells from each of the groups were seeded into 96-well culture plates. Next, the cells were incubated in 200 μl DMEM and 20 μl MTT solution (5 mg/ml) at 37°C for 4 h. The MTT formazan product was then solubilized in acidic isopropanol in each well, and the absorbance values for each sample were measured at 546 nm using a microplate reader (PR 3100 TSC Microplate Reader, Bio-Rad Laboratories, Inc.). Survival curves were constructed according to the results of the MTT assay.

### Cellular apoptosis assessment by flow cytometry

The assessment of cellular apoptosis was performed using the Annexin V-FITC-propidium iodide (PI) kit and the BD FACSCanto flow cytometer (BD Biosciences, Franklin Lakes, NJ, USA) 72 h after the co-transfection of the cells. Firstly, the cells were trypsinized and the density adjusted to 5×10^5^–1×10^6^ cells/ml. Next, the cells were washed three times in ice-cold PBS and centrifuged at 300 × g at 4°C for 10 min. Following the addition of 200 μl buffer, the cells were resuspended, and 5 μl Annexin V-FITC and 5 μl PI intermix were added. The reaction was conducted at room temperature for 15 min in the absence of light. Finally, 300 μl buffer was added to the samples, which were immediately assessed by flow cytometry.

### Statistical analysis

The data are presented as the mean ± standard deviation. A one-way analysis of variance and Student-Newman-Keuls tests were used. The statistical analyses were performed using SPSS 15.0 statistical software (SPSS, Inc., Chicago, IL, USA). P<0.05 was considered to indicate a statistically significant difference.

## Results

### Transcriptional expression of Grp78 and Grp94 in SGC-7901 cells

**Figure 1:**
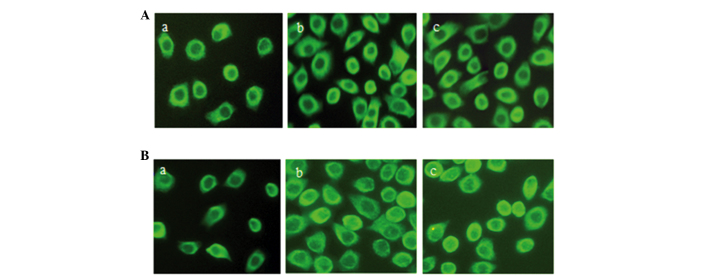
Immunofluorescence analysis revealing the expression of Grp78 and Grp94 in SGC-7901 cells. (A) Expression of Grp78 susbequent to stable transfection in the (a) experimental, (b) negative control and (c) blank control groups. (B) Expression of Grp94 subsequent to stable transfection in the (a) experimental, (b) negative control and (c) blank control groups. Magnification, ×200.

The mRNA levels of Grp78 and Grp94 in the SGC-7901 cells from the experimental and negative control groups were detected 72 h after stable transfection and then quantified by indirect immunofluorescence assay. The mRNA levels of Grp78 and Grp94 in the cells from the experimental group were 0.49±0.02 and 0.41±0.01, respectively. These values were less (P<0.001) than those observed from the negative control (1.01±0.02 and 1.00±0.02) and blank control (1.01±0.02 and 1.00±0.02) groups. The results revealed that the co-transfection of the SGC-7901 cells with Grp78 and Grp94 recombinant expression vectors reduced the relative expression levels of Grp78 and Grp94, respectively. Following stable transfection, the mean level of Grp78-positive cells in the experimental group (29.33%) was significantly reduced (P<0.001) compared with the negative (89.33%) and blank (80.25%) control groups (Fig. 1A). There was no significant difference reported between the level of Grp78-positive cells in the negative and blank control groups (Fig. 1A).

A similar pattern was observed for Grp94-positive cells. The mean frequency of positive cells in the experimental group (31.17%) was significantly reduced (P<0.001) following stable transfection compared with the negative (86.14%) and blank (78.53%) control groups. There was no significant difference identified between the level of Grp78-positive cells in the negative and blank control groups (Fig. 1B).

### MTT assay and survival curves

**Figure 2:**
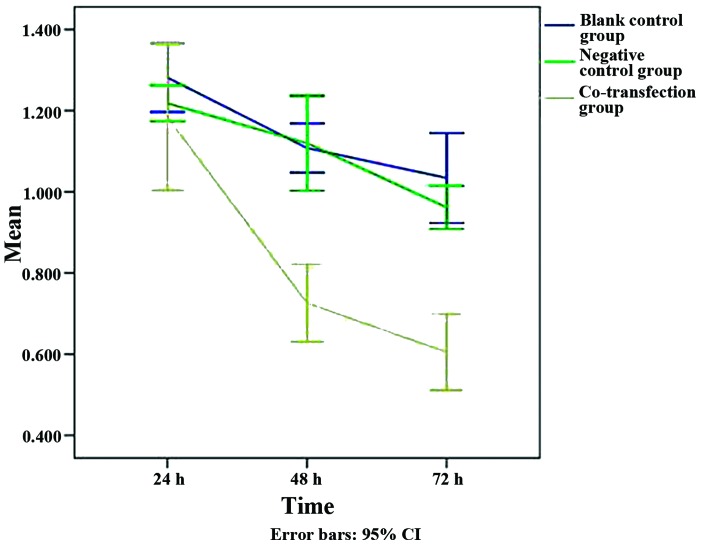
Survival rates of the cells in the experimental, negative control and blank control groups decreased over time following transfection. At 24 h post-transfection, no significant differences were identified between the groups. However, at 48 and 72 h post-transfection, the experimental group demonstrated a significant decrease in survival rate compared with the negative and blank control groups. CI, confidence interval.

The MTT assay, which was used to analyze the rate of cell growth and metabolism, revealed that the survival rate of the cells in the three groups decreased over time following transfection (Fig. 2). There were no evident differences identified between the rates of cellular survival in the groups at 24 h post-transfection (experimental group, 1.18±0.11; negative control group, 1.22±0.03; and blank control group, 1.28±0.05). However, the decrease in the rate of cellular proliferation at 48 h (0.73±0.06) and 72 h (0.61±0.06) after transfection differed in the experimental group.

At the 48- and 72-h time-points, the survival rate of the cells in the experimental group was significantly lower than that of the negative control group (1.12±0.07 and 0.96±0.03) and the blank control group (1.11±0.04 and 1.03±0.06) (Fig. 2; P<0.05). These results suggest that co-transfection effectively inhibited the rate of cell survival. During the 72-h incubation period, the rate of cellular survival decreased dramatically in the co-transfection group. There was no significant difference identified between the rates of cellular survival in the negative and blank control groups (Fig. 2).

### Cellular apoptosis, as determined by flow cytometry

**Figure 3:**
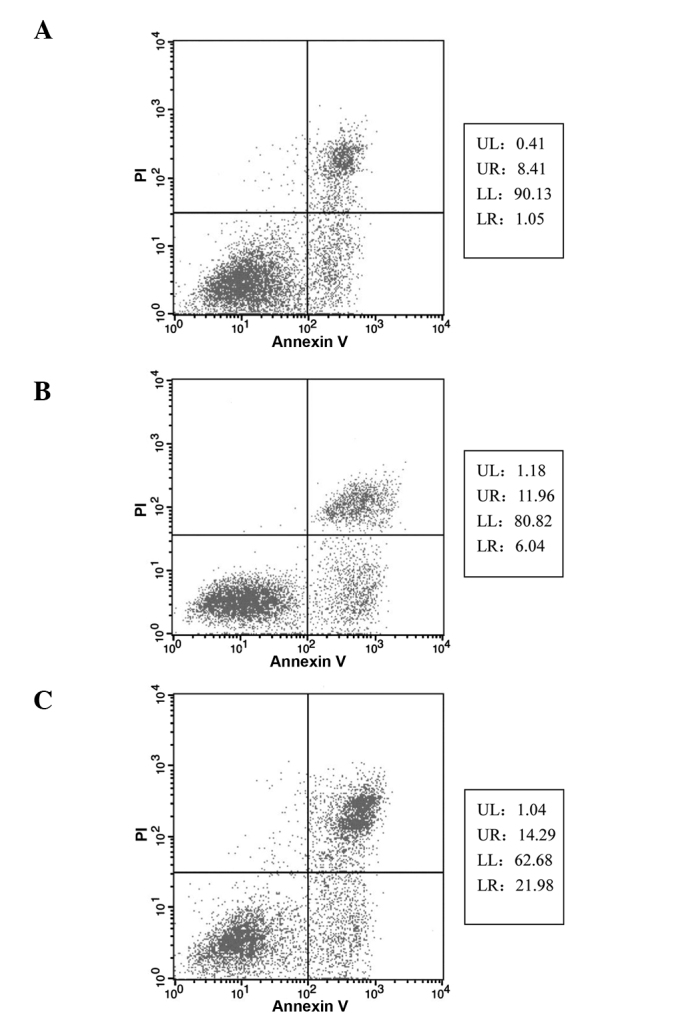
Flow cytometry analysis revealing the cellular apoptosis ratios at 72 h post-transfection in the (A) blank control, (B) negative control and (C) experimental groups. The apoptosis ratio of the co-transfected, experimental group (21.98%) was significantly higher than that of the blank (1.05%) and negative (6.04%) control groups. FITC, fluorescein isothiocyanate; PI, propidium iodide; PE, phycoerythrin; UL, upper left; UR, upper right; LL, lower left; LR, lower right.

At 72 h post-transfection, the cellular apoptosis ratios were quantified. The apoptosis ratio of the blank control group was low (1.05%). The ratio of the negative control group (6.04%) was higher compared with that of the blank control group, although this difference was not identified to be statistically significant. The apoptosis ratio of the co-transfected, experimental group (21.98%) was significantly higher than that of the control groups (P<0.05) (Fig. 3).

## Discussion

The mammalian stress response is an evolutionarily conserved mechanism, which enables cells to respond to adverse environmental or metabolic conditions (19). The response at the molecular level is characterized by the UPR in the ER. This response can synthesize specific sets of cellular proteins, which have protective functions for cellular survival (20). The most important functional proteins involved in the UPR are the Grps, which can be synthesized by the ER. The ER is an organelle where protein folding occurs, and where proteins attain a final conformational structure without any excessive misfolding or aggregation. When unfolded proteins accumulate in the ER, the homeostatic UPR is activated (21,22). The primary targets of the UPR are molecular chaperones and folding enzymes located in the ER, such as Grp78 and Grp94.

The activation of these proteins increases the capacity of the protein folding system, which can then maintain homeostasis within the ER and perform functional anti-apoptotic activities (23,24). Previous studies have indicated that the UPR is important for the quality control and proofreading of proteins within the ER. This process occurs not only under normal growth conditions, but also following the initiation of environmental stress (25,26). Although Grp overexpression has been demonstrated to protect cells exposed to ER stressors, the anti-apoptotic functions of these proteins in neoplastic cells could lead to cancer progression and chemotherapy resistance (19). Previous studies have revealed that the levels of Grp78 and Grp94 are elevated in a number of cancer cell lines, solid tumors and human cancer biopsies (3,4,6,8). These elevated levels are believed to be associated with malignancy.

Our previous studies reported that the levels of Grp78 and Grp94 were elevated in human gastric cancer specimens (17,18). Other studies have confirmed this result (27,28). For the present study, eukaryotic RNA interference expression vectors, specific for Grp78 and Grp94, were constructed in order to analyze the effect of Grp78 and Grp94 silencing upon the human gastric cancer SGC-7901 cell line (17,18). The results revealed that the expression of Grp78 mRNA and protein was significantly decreased following transfection with psiSTRIKE/Grp78. Similarly, the expression of Grp94 mRNA and protein was downregulated by transfection with psiSTRIKE/Grp94. Therefore, it was hypothesized that a decrease in the expression of Grps may inhibit the progression to late-stage gastric cancer, and may increase the sensitivity of cancer cells to chemotherapy.

The results revealed that at 72 h post-transfection, the expression of Grp78 and Grp94 was significantly downregulated in the experimental group compared with the control groups. Considering the effect of the transfection reagent Lipofectamine 2000, a group of cells that only received an equal dose of Lipofectamine 2000 was established. The cellular proliferation levels of the negative control group were lower than those of the blank control group, which indicated that the reagent may have affected the proliferation rate of the cells. However, no statistical difference was identified between these two groups, which suggested that Lipofectamine 2000 did not affect the results of the experiment. The results of the flow cytometry analysis revealed that at 72 h post-transfection, the number of apoptotic cells in the experimental group had notably increased. This increase was statistically significant compared with the control groups. No significant difference between the number of apoptotic cells in each control group was identified (6.04 vs. 1.05%).

The difference between the present study and previous studies was that the expression of Grp78 and Grp94 was simultaneously suppressed by co-transfection. The majority of previous studies had targeted the functional expression of Grp78. However, the present study hypothesized that Grp94 could be another important factor associated with the pathogenesis and progression of gastric cancer. Therefore, it was predicted that simultaneously downregulating the expression of Grp78 and Grp94 could inhibit the proliferation of the gastric cancer cell line. Due to limited time and funding constraints, the present study did not analyze all time-points. However, the selected time-points were considered sufficient in order to represent key alterations in the transcriptional and translational expression of Grps, and to identify differences in expression levels between the groups.

In conclusion, the *in vitro* co-transfection of SGC-7901 cells with PsiSTRIKE/Grp78 and psiSTRIKE/Grp94 significantly reduced the protein expression levels of Grp78 and Grp94. Furthermore, the *in vitro* co-transfection of SGC-7901 cells also inhibited cellular proliferation and increased the ratio of apoptosis. Future studies should attempt to use identical or similar methodological approaches to study the effect of co-transfection on gastric cell behavior *in vivo*.
